# Mental Health in Coronary Heart Disease (CHD) Patients: Findings from the UK Household Longitudinal Study (UKHLS)

**DOI:** 10.3390/healthcare11101364

**Published:** 2023-05-09

**Authors:** Weixi Kang, Antonio Malvaso

**Affiliations:** 1 UK DRI Care Research and Technology Centre, Department of Brain Sciences, Imperial College London, London SW7 2BX, UK; 2 Department of Brain and Behavioral Sciences, University of Pavia, 27100 Pavia, Italy

**Keywords:** mental health, GHQ-12, coronary heart disease

## Abstract

Objectives: Mental health conditions in patients with coronary heart disease (CHD) are closely related to clinical outcomes. Thus, this study’s goal is to investigate how CHD affects general and specific aspects of mental health. Methods: We analyzed data from Wave 10 Understanding Society: the UK Household Longitudinal Study (UKHLS), which were collected between 2018 and 2019. After removing people who had missing data, there were 450 participants who indicated that they have CHD, and 6138 age- and sex-matched healthy participants indicated that they were not clinically diagnosed with CHD. Results: The main findings were that participants with CHD had more mental health problems, as shown by the GHQ-12 summary score (t (449) = 6.00, *p* < 0.001, 95% C.I. [0.20, 0.40], Cohen’s d = 0.30), social dysfunction and anhedonia, (t (449) = 5.79, *p* < 0.001, 95% C.I. [0.20, 0.40], Cohen’s d = 0.30), depression and anxiety (t (449) = 5.04, *p* < 0.001, 95% C.I. [0.15, 0.33], Cohen’s d = 0.24), and loss of confidence (t (449) = 4.46, *p* < 0.001, 95% C.I. [0.11, 0.30], Cohen’s d = 0.21). Conclusion: This study implies that GHQ-12 is a valid assessment of mental health problems in CHD patients, and there is a need to consider how different aspects of mental health are affected by CHD rather than solely focusing on depression or anxiety problems alone in patients with CHD.

## 1. Introduction

The main cause of mortality globally is coronary heart disease (CHD) [1], which refers to conditions that occur when there is a buildup of plaque in the coronary arteries that supply blood to the heart muscle. This plaque buildup can cause the arteries to become narrow, reducing blood flow to the heart and increasing the risk of a heart attack. CHD puts a significant financial strain on the healthcare system [2]. Patients with CHD are more likely to develop mental health issues [3,4], including anxiety and depression [5,6], which have both been highly linked to poor outcomes in CHD patients [7,8,9,10]. These mental health conditions may not only increase the use of healthcare resources but can also result in disease deterioration [11].

Among commonly studied mental health conditions, including depression and anxiety, as pointed out by Carney and Freedland [12], several investigations have found that severe depression or clinically significant depression is common among patients with CHD. One study found that, among hospitalized patients with cardiac disease, 40.0% had clinically significant depression, as measured by a PHQ-9 score greater than 9. However, the prevalence of non-minimal depression (PHQ-9 score of 5 or greater) was even higher, at 78.4% [13]. Additionally, 13.6% CHD patients suffer from depression [14]. In comparison, only 3.8% of the general population suffers from depression, according to the WHO [15]. Similarly, anxiety is also very common among CHD patients. The prevalence of any anxiety illness is believed to be between 40% and 70% among CHD patients [16,17].

There are a few potential psychophysiological pathways that might explain the association between CHD and worse mental health. The first factor that may contribute to such an association might be the lifestyle factor. For instance, the Heart and Soul study [18,19,20] investigated the contribution of depression to the incidence of subsequent cardiovascular events in 1017 CHD patients. The results suggested that the association between mental health problems and cardiovascular events can be explained by factors such as physical inactivity, nonadherence to medication, and other behavioral factors of mental health. One possibility is inflation, as people with mental health problems are characterized by a sustained inflammatory state [21,22,23], and increased concentrations of a variety of inflammatory markers may have an important role in mediating the association between mental health and CHD [21,22,23]. Finally, endothelial dysfunction is linked to the majority of conventional cardiac risk factors that may be diagnosed in the early preclinical stages of atherosclerosis. Nitric oxide is normally produced by the vascular endothelium to maintain vascular tone and prevent smooth muscle cell proliferation, leukocyte adhesion, and platelet aggregation. Endothelial dysfunction may happen when the amount of endothelial nitric oxide is diminished, allowing the atherosclerotic process to proceed unabated [24]. Even in the absence of additional cardiac risk factors, evidence suggests that poor mental health is related to endothelial dysfunction [25,26,27,28,29].

The psychometric properties of the GHQ-12 have been examined in the literature [30,31,32,33,34,35]. Moreover, research suggests that the GHQ-12 is characterized by good specificity, reliability, and sensitivity [36,37]. Even though GHQ-12 was designed to be a unidimensional measure of mental health, there is debate about whether it should be utilized on a unidimensional or multidimensional scale. Furthermore, there is a lot of evidence for the GHQ-12 three-factor model among other factor solutions [38,39,40,41,42,43,44], including social dysfunction and anhedonia, depression and anxiety, and loss of confidence. The strong correlation between these components [30,40,42] is a common argument used to support the usage of the unidimensional GHQ-12 over the factor solution. Recent research utilizing simulated data, however, has shown that imposing a simplistic framework may artificially exaggerate correlations between modeled elements [45]. As a result, Griffith and Jones [46] warned that “taking these correlations as justification for unidimensionality risks a self-fulfilling prophecy of simplicity begetting simplicity” (p. 3). Given these disagreements, we investigated how the GHQ-12 unidimensional and multidimensional structures are affected by CHD.

While earlier research looked at how CHD affects mental health, with a major focus on sadness and anxiety, far less is understood about how CHD affects the general and specific aspects of mental health. As a result, we aimed to evaluate these CHD effects.

## 2. Methods

### 2.1. Data

For the present study, data from Wave 10 of the UK Household Longitudinal Study (UKHLS) [47], conducted between 2018 and 2019, were utilized. The initial sample included participants who self-reported having CHD (n = 450), while the remaining sample of age- and sex-matched individuals (n = 6138) reported no clinical diagnosis of CHD after the exclusion of those with missing data. The ratio of CHD participants and healthy people was slightly higher than previous studies (i.e., [48]), which could be explained by the missing data exclusion.

### 2.2. Measures

#### 2.2.1. CHD

The reliability of self-reported cardiovascular disease has been established in prior research (e.g., [49]). In the present study, participants were asked to respond to the following question as a means of indicating whether they had coronary heart disease: “Has a doctor or other health professional ever told you that you have any of these conditions? Coronary heart disease.” The response options included “Yes/No/Don’t know”. Participants who answered “Don’t know” were removed from further analysis.

#### 2.2.2. Mental Health

In this study, mental health was assessed using the GHQ-12, a unidimensional measure consisting of 12 items [50]. Responses on the Likert scale ranged from 0 (“Not at all”) to 3 (“Much more than usual”). A summary score across all 12 items was calculated to indicate their overall mental health, with higher scores indicating poorer mental health. The GHQ-12 was rated on a scale of 1 (“Not at all”) to 4 (“Much more than usual”) for the purpose of a factor analysis.

#### 2.2.3. Demographic Variables

Age, sex, monthly income, education, marital status, and residence were all demographic variables in the linear models.

### 2.3. Analysis

#### 2.3.1. Factor Model

In the present study, a confirmatory factor analysis (CFA) with oblique rotation was performed on the General Health Questionnaire-12 (GHQ-12) using MATLAB 2018 software. The analysis was conducted with a prespecified three-factor structure, which included social dysfunction and anhedonia, depression and anxiety, and loss of confidence. Subsequently, both the GHQ-12 summary score and component scores were standardized with a mean of 0 and a standard deviation of 1 for further analysis. This was done to ensure differences between groups were presented in Cohen’s d units.

#### 2.3.2. Linear Models

In this study, a predictive normative modeling approach was utilized to control for the impact of unbalanced factors on mental health between healthy individuals and those with CHD, such as demographics. Firstly, four generalized linear models were constructed using demographics as predictors and the GHQ-12 summary scores, social dysfunction and anhedonia, depression and anxiety, and loss of confidence as the dependent variables. These models were based on data from individuals who did not report having CHD. Secondly, the CHD patients’ demographics were incorporated into the models to predict their anticipated GHQ-12 summary scores, social dysfunction and anhedonia, depression and anxiety, and loss of confidence. Finally, one-sample *t*-tests were performed to assess the differences between the actual and predicted scores.

## 3. Results

Table 1 displays the descriptive statistics. The CFA produced three interpretable factors, including social dysfunction and anhedonia, depression and anxiety, and loss of confidence. Table 2 shows the factor loadings for each question.

The current study found the main effects of age (F(1, 6131) = 39.95, *p* < 0.001, η^2^ = 0.01), sex (F(1, 6131) = 58.36, *p* < 0.001, η^2^ = 0.01), monthly income (F(1, 6131) = 32.22, *p* < 0.001, η^2^ = 0.01), and legal marital status (F(1, 6131) = 143.56, *p* < 0.001, η^2^ = 0.00) on the GHQ-12 summary scores. Similarly, there was the main effects of sex (F(1, 6131) = 8.91, *p* < 0.01, η^2^ = 0.00), monthly income (F(1, 6131) = 14.18, *p* < 0.001, η^2^ = 0.00), and legal marital status (F(1, 6131) = 13.02, *p* < 0.001, η^2^ = 0.00) on social dysfunction and anhedonia. Moreover, there was the main effects of age (F(1, 6131) = 97.85, *p* < 0.001, η^2^ = 0.02), sex (F(1, 6131) = 95.06, *p* < 0.001, η^2^ = 0.02), monthly income (F(1, 6131) = 15.13, *p* < 0.001, η^2^ = 0.00), and legal marital status (F(1, 6131) = 11.00, *p* < 0.001, η^2^ = 0.00) on depression and anxiety. Finally, there was the main effects of age (F(1, 6131) = 23.73, *p* < 0.001, η^2^ = 0.00), sex (F(1, 6131) = 20.56, *p* < 0.001, η^2^ = 0.00), monthly income (F(1, 6131) = 49.77, *p* < 0.001, η^2^ = 0.01), and legal marital status (F(1, 6131) = 27.60, *p* < 0.001, η^2^ = 0.00) on loss of confidence. However, the main effect of age was not significant.

**Figure 1:**
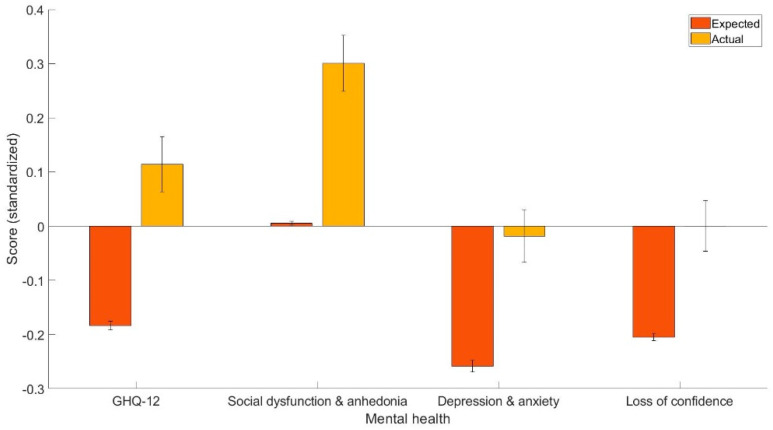
The expected and actual scores (standardized) for GHQ-12, social dysfunction and anhedonia, depression and anxiety, and loss of confidence.

The main finding was that CHD patients have worse overall mental health, as indicated by the GHQ-12 summary scores (t (449) = 6.00, *p* < 0.001, 95% C.I. [0.20, 0.40], Cohen’s d = 0.30), social dysfunction and anhedonia (t (449) = 5.79, *p* < 0.001, 95% C.I. [0.20, 0.40], Cohen’s d = 0.30), depression and anxiety (t (449) = 5.04, *p* < 0.001, 95% C.I. [0.15, 0.33], Cohen’s d = 0.24), and loss of confidence (t (449) = 4.46, *p* < 0.001, 95% C.I. [0.11, 0.30], Cohen’s d = 0.21). Figure 1 shows the means and standard errors of the projected and actual standardized scores.

## 4. Discussion

The current study sought to evaluate how CHD affects the general and specific aspects of mental health. By using a CFA and predictive normative modeling approach, the current study (1) identified the underlying factors of the GHQ-12, including social dysfunction and anhedonia, depression and anxiety, and loss of confidence, and (2) found that the patients had more overall mental health problems, as well as dysfunction and anhedonia, depression and anxiety, and loss of confidence.

In the current study, the CFA generated three factors labeled as social dysfunction and anhedonia, depression and anxiety, and loss of confidence. The current study’s three-factor structure solution is substantially compatible with prior research that identified three components in GHQ-12 [38,39,40,41,42,43,44]. Furthermore, as indicated in Table 1, the factor loadings in our investigation appeared to be high.

The result that CHD has a negative impact on patients’ overall mental health is mostly consistent with the assumption that mental health commodities are relatively frequent in patients. Furthermore, CHD patients had more social dysfunction and anhedonia, which is a novel finding, given that previous research has shown that anhedonia can independently predict patients’ health and more somatic and cognitive symptoms, as well as the combined endpoint of adverse clinical events and mortality one year after an acute coronary syndrome [51] and in patients following coronary artery stent implantation [52]. This result is also consistent with one previous study that found heart failure to be associated with poor psychosocial function [53]. In addition, these associations were still held after taking into account depression and the severity of depression. The finding that CHD patients have worse depression and anxiety is also consistent with previous studies that found the prevalence of depression and anxiety higher than the general population prevalence (see [12,54] for reviews). Finally, the current study also found that CHD patients have worse confidence compared with people who do not have CHD, which is of particular importance given that confidence in patients is closely related to clinical outcomes. Moreover, the effect sizes of the different dimensions of mental health were different, which may imply that the dimensions of mental health are affected by CHD differently; thus, there is a need to look at the specific dimensions of mental health in addition to general mental health.

Despite the present study’s advantages, there were several drawbacks. First, because the current study was cross-sectional, it could not demonstrate a causal link, because the interaction between CHD and mental health problems might be completely bidirectional. To demonstrate the temporal order, future research should take a longitudinal approach. Second, our study relied on self-reported measures, which are susceptible to self-reported bias. Future research may need to employ more objective metrics, such as clinical assessments, to determine whether the present findings are still valid. Finally, our study only included individuals from the UK, making it difficult to extrapolate the current findings to other countries.

## 5. Conclusions

To summarize, by using a CFA and predictive normative modeling approach, the current study replicated the three underlying structures of the GHQ-12 and found that the GHQ-12 summary scores revealed that patients have more overall mental health issues (Cohen’s d = 0.30), social dysfunction and anhedonia (Cohen’s d = 0.30), depression and anxiety, (Cohen’s d = 0.24), and loss of confidence (Cohen’s d = 0.21). This study suggests that GHQ-12 is a meaningful measure of mental health problems in CHD patients and that there is a need to investigate how different aspects of mental health are affected by CHD rather than focusing primarily on depression or anxiety difficulties in CHD patients. Clinicians should develop interventions that improve mental health in people with CHD, which can then lead to better outcomes.

## Figures and Tables

**Table 1:** The demographic characteristics for the healthy controls and CHD patients.

	Healthy Controls		CHD Patients	
	Mean	S.D.	Mean	S.D.
Age	68.47	8.22	68.29	11.60
Income (monthly)	£1832.58	£1570.07	£1699.32	£1170.78
	N	%	N	%
**Sex**				
Male	4167	67.89	308	68.44
Female	1971	32.11	142	31.56
**Education**				
Below college	4330	70.54	351	78.00
College	1808	29.46	99	22.00
**Legal marital status**				
Single	2048	33.37	178	39.56
Married	4090	66.63	272	60.44
**Residence**				
Urban	4348	70.84	323	71.78
Rural	1790	29.16	127	28.22

**Table 2:** The factor loadings for the three-factor structure of the GHQ-12.

GHQ-12 Items	Social Dysfunction and Anhedonia	Depression and Anxiety	Loss of Confidence
Concentration	**0.53**	0.25	−0.10
Loss of sleep	0.00	**0.66**	0.05
Playing a useful role	**0.67**	−0.16	0.16
Constantly under strain	**0.79**	−0.14	0.00
Problem overcoming difficulties	−0.01	**0.88**	−0.07
Unhappy or depressed	0.08	**0.56**	0.21
Losing confidence	**0.68**	0.22	−0.125
Believe worthless	**0.71**	−0.03	0.04
General happiness	0.013	**0.53**	0.34
Capable of making decisions	0.00	0.23	**0.70**
Ability to face problems	0.10	0.02	**0.74**
Enjoy day-to-day activities	**0.56**	0.10	0.10

## Data Availability

Publicly available datasets were analyzed in this study. This data can be found here: https://www.understandingsociety.ac.uk (accessed on 7 February 2022).

## References

[1] Wang H., Naghavi M., Allen C., Barber R.M., Bhutta Z.A., Carter A., Li Y., Wang L., Liu Y., Yin P. (2016). Global, regional, and national life expectancy, all-cause mortality, and cause-specific mortality for 249 causes of death, 1980–2015: A systematic analysis for the Global Burden of Disease Study 2015. Lancet.

[2] Hanna I.R., Wenger N.K. (2005). Secondary prevention of coronary heart disease in elderly patients. Am. Fam. Physician.

[3] Chaddha A., Robinson E.A., Kline-Rogers E., Alexandris-Souphis T., Rubenfire M. (2016). Mental health and cardiovascular disease. Am. J. Med..

[4] Gostoli S., Buzzichelli S., Guidi J., Sirri L., Marzola E., Roncuzzi R., Abbate-Daga G., Fava G.A., Rafanelli C. (2023). An innovative approach to the assessment of mood disturbances in patients with acute coronary syndrome. CNS Spectr..

[5] Vaccarino V., Badimon L., Bremner J.D., Cenko E., Cubedo J., Dorobantu M., Duncker D.J., Koller A., Manfrini O., Milicic D. (2019). Depression and coronary heart disease: 2018 position paper of the ESC working group on coronary pathophysiology and microcirculation. Eur. Heart J..

[6] Bashiri Z., Aghajani M., Alavi N.M. (2016). Effects of psychoeducation on mental health in patients with coronary heart disease. Iran. Red Crescent Med. J..

[7] Ono M., Serruys P.W., Garg S., Kawashima H., Gao C., Hara H., Lunardi M., Wang R., O’leary N., Wykrzykowska J.J. (2022). Effect of patient-reported preprocedural physical and mental health on 10-year mortality after percutaneous or surgical coronary revascularization. Circulation.

[8] Mayer O., Bruthans J., Seidlerová J., Karnosová P., Mateřánková M., Gelžinský J., Rychecká M., Cífková R., Filipovský J. (2020). Mood disorders impaired quality of life but not the mortality or morbidity risk in stable coronary heart disease patients. Acta Cardiol..

[9] Fan Y., Ho M.R., Shen B. (2023). Loneliness predicts physical and mental health-related quality of life over 9 months among patients with coronary heart disease. Appl. Psychol. Health Well Being.

[10] Barth J., Schumacher M., Herrmann-Lingen C. (2004). Depression as a risk factor for mortality in patients with coronary heart disease: A meta-analysis. Psychosom. Med..

[11] Dehdari T., Heidarnia A., Ramezankhani A., Sadeghian S., Ghofranipour F. (2009). Effects of progressive muscular relaxation training on quality of life in anxious patients after coronary artery bypass graft surgery. Indian J. Med. Res..

[12] Carney R.M., Freedland K.E. (2017). Depression and coronary heart disease. Nat. Rev. Cardiol..

[13] Bahall M. (2019). Prevalence and associations of depression among patients with cardiac diseases in a public health institute in Trinidad and Tobago. BMC Psychiatry.

[14] Haddad M., Walters P., Phillips R., Tsakok J., Williams P., Mann A., Tylee A. (2013). Detecting depression in patients with coronary heart disease: A diagnostic evaluation of the PHQ-9 and HADS-D in primary care, findings from the UPBEAT-UK study. PLoS ONE.

[15] Organisation W.H. (2021). Depression. https://www.who.int/news-room/fact-sheets/detail/depression.

[16] Gu G., Zhou Y., Zhang Y., Cui W. (2016). Increased prevalence of anxiety and depression symptoms in patients with coronary artery disease before and after percutaneous coronary intervention treatment. BMC Psychiatry.

[17] Silarova B., Nagyova I., Van Dijk J.P., Rosenberger J., Reijneveld S.A. (2014). Anxiety and sense of coherence in Roma and non-Roma coronary heart disease patients. Ethn. Health.

[18] Cohen B.E., Panguluri P., Na B., Whooley M.A. (2010). Psychological risk factors and the metabolic syndrome in patients with coronary heart disease: Findings from the Heart and Soul Study. Psychiatry Res..

[19] Ruo B., Rumsfeld J.S., Hlatky M.A., Liu H., Browner W.S., Whooley M.A. (2003). Depressive symptoms and health-related quality of life: The Heart and Soul Study. JAMA.

[20] Whooley M.A., De Jonge P., Vittinghoff E., Otte C., Moos R., Carney R.M., Sadia A., Sunaina D., Beeya N., Feldman M.D. (2008). Depressive symptoms, health behaviors, and risk of cardiovascular events in patients with coronary heart disease. JAMA.

[21] Currier M.B., Nemeroff C.B. (2010). Inflammation and mood disorders: Proinflammatory cytokines and the pathogenesis of depression. Anti Inflamm. Anti Allergy Agents Med. Chem..

[22] Nemeroff C.B., Goldschmidt-Clermont P.J. (2012). Heartache and heartbreak—The link between depression and cardiovascular disease. Nat. Rev. Cardiol..

[23] Raedler T.J. (2011). Inflammatory mechanisms in major depressive disorder. Curr. Opin. Psychiatry.

[24] Shimokawa H. (1999). Primary endothelial dysfunction: Atherosclerosis. J. Mol. Cell. Cardiol..

[25] Broadley A.J.M., Korszun A., Jones C.J.H., Frenneaux M.P. (2002). Arterial endothelial function is impaired in treated depression. Heart.

[26] Cooper D.C., Tomfohr L.M., Milic M.S., Natarajan L., Bardwell W.A., Ziegler M.G., Dimsdale J.E. (2011). Depressed mood and flow-mediated dilation: A systematic review and meta-analysis. Psychosom. Med..

[27] Rajagopalan S., Brook R., Rubenfire M., Pitt E., Young E., Pitt B. (2001). Abnormal brachial artery flow-mediated vasodilation in young adults with major depression. Am. J. Cardiol..

[28] Rybakowski J.K., Wykretowicz A., Heymann-Szlachcinska A., Wysocki H. (2006). Impairment of endothelial function in unipolar and bipolar depression. Biol. Psychiatry.

[29] Sherwood A., Hinderliter A.L., Watkins L.L., Waugh R.A., Blumenthal J.A. (2005). Impaired endothelial function in coronary heart disease patients with depressive symptomatology. J. Am. Coll. Cardiol..

[30] El-Metwally A., Javed S., Razzak H.A., Aldossari K.K., Aldiab A., Al-Ghamdi S.H., Househ M., Mamdouh M.S., Al-Zahrani J.M. (2018). The factor structure of the general health questionnaire (GHQ12) in Saudi Arabia. BMC Health Serv. Res..

[31] Fernandes H.M., Vasconcelos-Raposo J. (2012). Factorial validity and invariance of the GHQ-12 among clinical and nonclinical samples. Assessment.

[32] Hankins M. (2008). The factor structure of the twelve-item general health questionnaire (GHQ-12): The result of negative phrasing?. Clin. Pract. Epidemiol. Ment. Health.

[33] López M.P.S., Dresch V. (2008). The 12-item general health questionnaire (GHQ-12): Reliability, external validity and factor structure in the Spanish population. Psicothema.

[34] Salama-Younes M., Montazeri A., Ismaïl A., Roncin C. (2009). Factor structure and internal consistency of the 12-item general health questionnaire (GHQ-12) and the subjective vitality scale (VS), and the relationship between them: A study from France. Health Qual. Life Outcomes.

[35] Smith A.B., Fallowfield L.J., Stark D.P., Velikova G., Jenkins V. (2010). A Rasch and confirmatory factor analysis of the general health questionnaire (GHQ)-12. Health Qual. Life Outcomes.

[36] Daradkeh T.K., Ghubash R., El-Rufaie O.E.F. (2001). Reliability, validity, and factor structure of the Arabic version of the 12-item general health questionnaire. Psychol. Rep..

[37] Endsley P., Weobong B., Nadkarni A. (2017). The psychometric properties of GHQ for detecting common mental disorder among community-dwelli men in Goa, India. Asian J. Psychiatr..

[38] Campbell A., Knowles S. (2007). A confirmatory factor analysis of the GHQ12 using a large Australian sample. Eur. J. Psychol. Assess..

[39] Gao W., Stark D., Bennett M.I., Siegert R.J., Murray S., Higginson I.J. (2011). Using the 12-item general health questionnaire to screen psychological distress from survivorship to end-of-life care: Dimensionality and item quality. Psycho Oncol..

[40] Graetz B. (1991). Multidimensional properties of the general health questionnaire. Soc. Psychiatry Psychiatr. Epidemiol..

[41] Martin L.M., Leff M., Calonge N., Garrett C., Nelson D.E. (2000). Validation of self-reported chronic conditions and health services in a managed care population. Am. J. Prev. Med..

[42] Padrón A., Galán I., Durbán M., Gandarillas A., Rodríguez-Artalejo F. (2011). Confirmatory factor analysis of the general health questionnaire (GHQ-12) in Spanish adolescents. Qual. Life Res..

[43] Penninkilampi-Kerola V., Miettunen J., Ebeling H. (2006). Health and disability: A comparative assessment of the factor structures and psychometric properties of the GHQ-12 and the GHQ-20 based on data from a Finnish population-based sample. Scand. J. Psychol..

[44] Rajabi G., Sheykhshabani S.H. (2009). Factor structure of the 12-item general health questionnaire. J. Educ. Psychol..

[45] Marsh H.W., Morin A.J., Parker P.D., Kaur G. (2014). Exploratory structural equation modeling: An integration of the best features of exploratory and confirmatory factor analysis. Annu. Rev. Clin. Psychol..

[46] Griffith G.J., Jones K. (2019). Understanding the population structure of the GHQ-12: Methodological considerations in dimensionally complex measurement outcomes. Soc. Sci. Med..

[47] University of Essex, Institute for Social and Economic Research (2022). Understanding Society: Waves 1-11, 2009-2020 and Harmonised BHPS: Waves 1–18, 1991–2009.

[48] Bhatnagar P., Wickramasinghe K., Wilkins E., Townsend N. (2016). Trends in the epidemiology of cardiovascular disease in the UK. Heart.

[49] Barr E.L., Tonkin A.M., Welborn T.A., Shaw J.E. (2009). Validity of self-reported cardiovascular disease events in comparison to medical record adjudication and a statewide hospital morbidity database: The AusDiab study. Intern. Med. J..

[50] Goldberg D., Williams P. (1988). A User’s Guide to the General Health Questionnaire.

[51] Davidson K.W., Burg M.M., Kronish I.M., Shimbo D., Dettenborn L., Mehran R., Vorchheimer D., Clemow L., Schwartz J.E., Rieckmann N. (2010). Association of anhedonia with recurrent major adverse cardiac events and mortality 1 year after acute coronary syndrome. Arch. Gen. Psychiatry.

[52] Denollet J., Pedersen S.S., Daemen J., De Jaegere P., Serruys P.W., Van Domburg R.T. (2008). Reduced positive affect (anhedonia) predicts major clinical events following implantation of coronary-artery stents. J. Intern. Med..

[53] López Castro J., Cid Conde L., Fernández Rodríguez V., Failde Garrido J.M., Almazán Ortega R. (2013). Analysis of quality of life using the generic SF-36 questionnaire in patients with heart failure. Rev. Calid. Asist. Organo Soc. Esp. Calid. Asist..

[54] Farquhar J.M., Stonerock G.L., Blumenthal J.A. (2018). Treatment of anxiety in patients with coronary heart disease: A systematic review. Psychosomatics.

